# Cell-type- and region-specific restriction of neurotropic flavivirus infection by viperin

**DOI:** 10.1186/s12974-018-1119-3

**Published:** 2018-03-15

**Authors:** Richard Lindqvist, Chaitanya Kurhade, Jonathan D. Gilthorpe, Anna K. Överby

**Affiliations:** 1 Department of Clinical Microbiology, Virology, Umeå University, 90185 Umeå, Sweden; 2 The Laboratory for Molecular Infection Medicine Sweden (MIMS), Umeå University, 90187 Umeå, Sweden; 3 Department of Pharmacology and Clinical Neurosciences, Umeå University, 90187 Umeå, Sweden

**Keywords:** Viperin, Interferon, Flavivirus, Neurons, Astrocytes

## Abstract

**Background:**

Flaviviruses are a group of diverse and emerging arboviruses and an immense global health problem. A number of flaviviruses are neurotropic, causing severe encephalitis and even death. Type I interferons (IFNs) are the first line of defense of the innate immune system against flavivirus infection. IFNs elicit the concerted action of numerous interferon-stimulated genes (ISGs) to restrict both virus infection and replication. Viperin (virus-inhibitory protein, endoplasmic reticulum-associated, IFN-inducible) is an ISG with broad-spectrum antiviral activity against multiple flaviviruses in vitro. Its activity in vivo restricts neurotropic infections to specific regions of the central nervous system (CNS). However, the cell types in which viperin activity is required are unknown. Here we have examined both the regional and cell-type specificity of viperin in the defense against infection by several model neurotropic flaviviruses.

**Methods:**

Viral burden and IFN induction were analyzed in vivo in wild-type and viperin^−/−^ mice infected with Langat virus (LGTV). The effects of IFN pretreatment were tested in vitro in primary neural cultures from different brain regions in response to infection with tick-borne encephalitis virus (TBEV), West Nile virus (WNV), and Zika virus (ZIKV).

**Results:**

Viperin activity restricted nonlethal LGTV infection in the spleen and the olfactory bulb following infection via a peripheral route. Viperin activity was also necessary to restrict LGTV replication in the olfactory bulb and the cerebrum following CNS infection, but not in the cerebellum. In vitro, viperin could restrict TBEV replication in primary cortical neurons, but not in the cerebellar granule cell neurons. Interferon-induced viperin was also very important in primary cortical neurons to control TBEV, WNV, and ZIKV.

**Conclusions:**

Our findings show that viperin restricts replication of neurotropic flaviviruses in the CNS in a region- and cell-type-specific manner. The most important sites of activity are the olfactory bulb and cerebrum. Activity within the cerebrum is required in the cortical neurons in order to restrict spread. This study exemplifies cell type and regional diversity of the IFN response within the CNS and shows the importance of a potent broad-spectrum antiviral ISG.

**Electronic supplementary material:**

The online version of this article (10.1186/s12974-018-1119-3) contains supplementary material, which is available to authorized users.

## Background

The type I interferon (IFN) response is the first line of defense against virus infections. IFNs are induced and secreted in infected cells upon pathogen recognition. Secreted type I IFN binds to the IFN-α/β receptors (IFNARs) in both an autocrine and a paracrine manner, activating the Janus kinase (JAK)/signal transducer and activator of transcription (STAT) pathway, which induces the expression of hundreds of interferon-stimulated genes (ISGs) [1]. ISG responses vary according to specific pathogens, the cell type infected, and the immune status of the host, as well as the immune evasion capabilities of the pathogen. Given that neurons are largely nonrenewable, neurotropic viruses need to be cleared from the CNS without causing excessive immune-mediated damage. We have shown that the local IFN response in the brain is capable of protecting mice from low doses of flavivirus [2, 3]. Astrocytes are a major source of IFNs, the secretion of which protects neighboring astrocytes as well as neurons very rapidly following infection [4]. Recent studies have also shown that viral tropism within the CNS is shaped by the immune response within specific brain regions [2, 5]. However, how innate antiviral responses in specific cell types and regions of the brain shape immune protection is not clear.

Viperin (virus inhibitory protein, endoplasmic reticulum-associated, interferon-inducible) is an ISG with broad-spectrum antiviral activity [6]. Viperin expression is low at the basal state. However, upon viral recognition, it is highly induced in an IFN-dependent as well as in an IFN-independent manner [7–9]. It has broad-range antiviral activity against influenza A virus [10, 11], human immunodeficiency virus [12], chikungunya virus [13], Sindbis virus [14], and respiratory syncytial virus [15]. It is also active against members of the *Flaviviridae* family such as hepatitis C virus (HCV), Zika virus (ZIKV), dengue virus (DENV), West Nile virus (WNV), and tick-borne encephalitis virus (TBEV) [16–21]. Despite its broad antiviral activity in vitro, the antiviral activity of viperin in vivo has only been demonstrated in WNV, respiratory syncytial virus, and Chikungunya virus infection [13, 15, 18]. One possible reason for this is that most viruses are able to block the IFN response in order to minimize ISG expression and the restriction of viral infection [22–26]. Another explanation could be redundancy among ISGs, where multiple ISGs act in a coordinated manner to mount an antiviral response. Interestingly, although low basal levels of viperin are found in most cell types, we have found high levels of viperin in the brain, specifically in primary astrocyte cultures in vitro [4]. They also show a rapid response to viral infection by upregulation of viperin expression. This could mean that viperin plays an important role in the antiviral defense of specific cell types. We have identified viperin as a potent inhibitor of TBEV [19], Langat virus (LGTV), [27] and ZIKV [28]. Since these are neurotropic viruses, they represent useful models to investigate the antiviral capacity of viperin in the brain.

Here we have used LGTV as a model for tick-borne flavivirus infection in vivo. We found that viperin specifically restricted replication in vivo in the olfactory bulb and in the cerebrum, but not in the cerebellum. In primary cells isolated from the cerebrum, both neurons and astrocytes relied on viperin for their antiviral response. However, viperin failed to restrict highly pathogenic TBEV replication in the granule cell neurons from the cerebellum. Furthermore, we found that viperin was required for the IFN-mediated antiviral responses against flaviviruses in cortical neurons. In summary, our findings show that viperin acts as an inhibitor of flaviviruses in the brain with a region-specific role in the olfactory bulb and cerebrum and cell-specific role in cortical neurons.

## Methods

### Viral infections of mice

C57BL/6 wild-type (WT) mice were purchased from Harlan laboratories/ENVIGO. Viperin^−/−^ mice on C57BL/6 background were a kind gift from Peter Cresswell, Department of Immunobiology, Yale University School of Medicine. In vivo infection experiments were performed at Umeå Center for Comparative Biology (UCCB) using LGTV strain TP21 (a kind gift from Gerhard Dobler, the Bundeswehr Institute of Microbiology, Munich, Germany). The virus was titrated by focus-forming assay on VeroB4 cells [29]. Six- to ten-week-old mice were infected intraperitoneally with the indicated focus-forming units (FFU) of LGTV in 100 μl phosphate buffered saline (PBS). For intracranial infections, mice were anesthetized by intraperitoneal injection with a mixture of ketamine (Intervet) (100 μg/g body weight) and xylazine (Bayer) (5 μg/g body weight), then injected with the indicated FFU of LGTV in 20 μl PBS. Survival was monitored for 21 days. Mice that lost more than 20% of their body weight were sacrificed. For organ harvesting, mice were perfused with 20 ml of PBS and organs were harvested in Trizol reagent for RNA extraction.

### Viral infections of cells

VeroB4 cells were cultured in Medium 199/Earle’s Balanced Salt Solution (EBSS) (HyClone) supplemented with 10% fetal bovine serum (FBS) (Thermo Fisher Scientific) and 0.2% penicillin and streptomycin (Gibco). Primary mouse astrocytes and cortical and cerebellar granule cell neurons were isolated and cultured as described [4, 30–32]. In brief, astrocytes were isolated from postnatal day 1–4 mice. Cerebral cortices were isolated in HBSS (Gibco) containing 0.5% penicillin and streptomycin (Gibco) and 0.4% glucose. Tissues were dissociated by pipetting and seeded in poly-d-lysine-coated T75 tissue culture flasks. Astrocytes were grown in Dulbecco’s modified Eagle’s medium (DMEM) supplemented with 10% FBS (Thermo Fisher Scientific), 0.2% penicillin and streptomycin (Gibco), and 2 mM L-glutamine (Gibco). Primary cortical neurons were isolated from the cerebral cortices of embryonic day 17, and cerebellar granule cell neurons from postnatal day 6 and 7 mice, and seeded onto poly-d-lysine-coated wells. Neurons were grown in neurobasal medium (Gibco) supplemented with B27 (Gibco), 0.2% penicillin and streptomycin, and 2 mM L-glutamine (Gibco). TBEV Hypr 71 (isolated in 1953 from patient blood in Czech Republic), LGTV TP21, and ZIKV MR 766 were kind gifts of Gerhard Dobler and were propagated in VeroB4 cells. JEV (Nakayama strain) and WNV (isolated in 2003 in Israel WNV_0304h_ISR00, passage number 5) were kind gifts from S. Vene (Folkhälsoinstitutet, Stockholm, Sweden).

### Virus titrations and infection

TBEV, LGTV, and ZIKV were titrated using focus-forming assays as previously described [4, 29]. In brief, VeroB4 cells were infected with 10-fold serial dilutions of TBEV, LGTV, WNV, JEV, or ZIKV. After 48 h of infection, cells were fixed with 4% formaldehyde and permeabilized in PBS containing 0.5% Triton X-100 and 20 mM glycine. Viral foci were detected using primary mouse antibodies directed against TBEV or flavivirus envelope protein (TBEV, 1493 1:1000 [33], LGTV 1786 1:1000 [33], ZIKV, WNV, JEV HB112 ATCC 1:1000 [34, 35]) followed by staining with a horseradish peroxidase-conjugated anti-mouse secondary antibody (1:2000, Thermo Fisher Scientific). Viral foci were detected using TrueBlue peroxidase substrate (KPL, Gaithersburg, MD). For in vitro infections, monolayers of cells were infected with 0.1 multiplicity of infection (MOI) of TBEV, LGTV, WNV, JEV, and ZIKV. After 1 h of infection, the inoculum was removed and replaced with growth medium. Experiments with TBEV, WNV, and JEV were performed in the biosafety level 3 facility at Umeå University.

### IFN-α ELISA

The amount of murine IFN-α in mouse serum was determined by enzyme-linked immunosorbent assay (ELISA) according to the manufacturer’s instructions (PBL).

### RNA isolation and qPCR

Mouse tissues were homogenized in Trizol reagent (Invitrogen) using Lysis Matrix (Nordic Biolabs) and a Fast Prep-24 tissue homogenizer (MP). RNA was extracted using the Nucleo-Spin RNA II kit (Macherey Nagel). Five hundred nanograms of total RNA was used to synthesize cDNA with the QuantiTect Reverse Transcription Kit (Qiagen). Expression levels of mouse GAPDH, IFN-β, IFN-α2, Mx1, IFIT1, and CXCL10 were detected by validated QuantiTect primer assays (Qiagen) and the KAPA SYBR FAST (Kapa Biosystems) quantitative polymerase chain reaction (qPCR) kit using a StepOnePlus fast real-time PCR system as previously described [2, 3]. TBEV and LGTV RNA were quantified using TaqMan probe and primers [2, 36] using KAPA PROBE FAST kit (Kapa Biosystems).

### IFN treatment and blockage of IFNAR

Astrocytes and cortical neurons were treated with 5000 U/ml IFNαB/D [37] 16 h before infection, and virus titers were determined by focus-forming assays. The IFNAR receptor was blocked using monoclonal antibodies as previously described [4]. In brief, cells were incubated for 2 h with either 10 μg/mL MAR1-5A3 (eBioscience, 16-5945-85 [38]) or IgGI κ isotype control (eBioscience 14-4714-85). After 2 h of incubation, the medium was removed and cells were infected for 1 h. After the infection, the inoculum was replaced with medium containing 10 μg/mL antibody.

### Western blotting

Cells were lysed, and samples were prepared for SDS-PAGE as described [19]. Samples were separated using pre-casted bolt 4–12% Bis-Tris gels (Invitrogen) followed by transfer to Immobilon-P PVDF membrane (Millipore). Membranes were stained as previously described [19]. Primary antibodies directed against TBEV E (mouse monoclonal 1493, 1:5000 [33]), actin (rabbit polyclonal, 1:2500 Sigma [4]), and viperin (mouse monoclonal, 1:500 Abcam [4]) were used.

### Statistical analyses

Data from cell culture experiments were analyzed using Graphpad Prism software. Unpaired *t* test was used to analyze qPCR data, Mann-Whitney test for in vivo experiments, and log-rank (Mantel-Cox) for survival analyses.

## Results

### Viperin restricts viral replication specifically in the olfactory bulb and prevents viral spread into the cerebrum

**Fig. 1:**
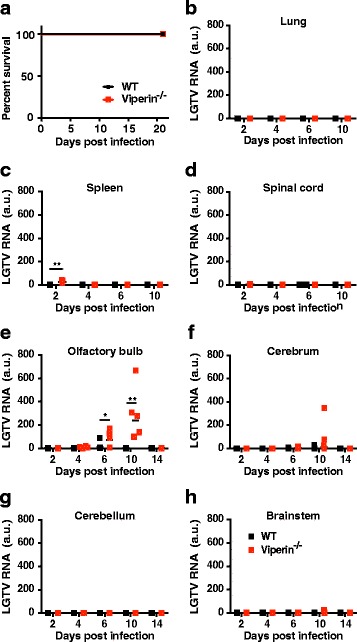
Viperin controls virus replication in the olfactory bulb. Survival analysis and viral burdens of LGTV infected WT and viperin^−/−^ mice after intraperitoneal inoculation. **a** Survival analysis of 6- to 8-week-old WT and vipeirn^−/−^ mice after inoculation of 10^4^ FFU of LGTV by intraperitoneal route (*n* = 10). Survival differences were tested for statistical significance by log-rank test. **b**–**h** Viral burdens in different organs (lung, spleen, spinal cord, olfactory bulb, cerebrum, cerebellum, and brain stem) of WT and Viperin^−/−^ mice after intraperitoneal infection of LGTV (10^4^ FFU, *n* = 5) measured by qPCR detecting LGTV NS3 gene (detection limit 10 copies) and normalized to intracellular GAPDH levels. Each data point represents an individual mouse. Bars indicated mean values. Asterisks indicate statistical significance calculated by the Mann-Whitney test (*P < 0.05; **P < 0.005). a.u., arbitrary units

We have shown previously that overexpression of viperin in vitro strongly reduces viral replication and the formation of progeny virus of highly pathogenic TBEV [19] and its low virulence model, LGTV [27]. To address the in vivo significance of viperin against tick-borne neurotropic flaviviruses, WT and viperin^−/−^ mice were infected intraperitoneally (ip) with 10^4^ FFU LGTV and bodyweight and survival was monitored. All mice survived the infections and did not display any overt signs of disease or loss in body weight (Fig. 1a and Additional file 1: Figure S1). To investigate the subclinical infection in different organs (lung, spleen, spinal cord, olfactory bulb, cerebrum, cerebellum, and brain stem), RNA was isolated and analyzed for viral burden at different days post-infection (dpi) (Fig. 1b–h). In WT mice, LGTV RNA was only detected in the olfactory bulb transiently at 6 dpi (Fig. 1e), indicating that the virus entered the brain but was cleared. However, in the absence of viperin, LGTV was first detected in the spleen at 2 dpi but was cleared by 4 dpi (Fig. 1b, c). Subsequently, a high viral burden was detected in the olfactory bulb at 6–10 dpi, and in the absence of viperin, LGTV disseminated into the cerebrum. However, the virus was cleared by 14 dpi in all organs (Fig. 1e, f). Hence, viperin is not necessary to protect the mice from neuroinvasion by LGTV, but it helps to limit the viral spread and mediate rapid clearance of the virus.

### Viperin promotes circulating IFNα production but not local IFN induction in the CNS

**Fig. 2:**
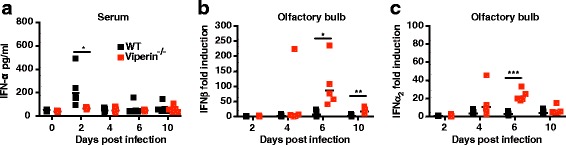
Viperin promotes the systemic IFNα response. **a** WT and viperin^−/−^ mice were infected intraperitoneally with 10^4^ FFU of LGTV. Serum samples were collected at indicated time points (*n* = 5). The IFNα protein level was measured by ELISA. **b**, **c** WT and viperin^−/−^ mice were infected intraperitoneally with 10^4^ FFU of LGTV. The olfactory bulb was collected at indicated time points (*n* = 5), and IFNβ and IFNα_2_ transcripts were detected using qPCR. Expression levels were normalized to the endogenous GAPDH expression and depicted as fold induction over uninfected. Each data point represents an individual mouse. Bars indicated mean values. Asterisks indicate statistical significance calculated by the Mann-Whitney test (*P < 0.05, **P < 0.005; ***P < 0.001)

Viperin has been shown to promote the IFN response in plasmacytoid dendritic cells [39]. Lowered IFN production in viperin^−/−^ mice could explain increased viral replication in the olfactory bulb and dissemination into the cerebrum. To assess the role of viperin in the IFN response, WT and viperin^−/−^ mice were infected with 10^4^ FFU of LGTV and levels of IFN-α were monitored in serum by ELISA (Fig. 2a). We observed a peak of IFNα at 2 dpi in WT mice before a return to basal levels at 4 dpi. No induction of IFNα was observed in the serum of viperin^−/−^ mice at any time point, indicating that viperin plays a role in IFN induction during LGTV infection. However, in the olfactory bulb, levels of IFNβ and IFNα_2_ mRNA were higher in viperin^−/−^ mice compared to WT, and IFN levels correlated well with viral replication (Fig. 2b, c). Thus, it appears that viperin expression is necessary for systemic, but not local, IFN production in the brain.

### Viperin protects mice from LGTV-induced neurovirulence by inhibiting viral replication in the olfactory bulb and cerebrum

**Fig. 3:**
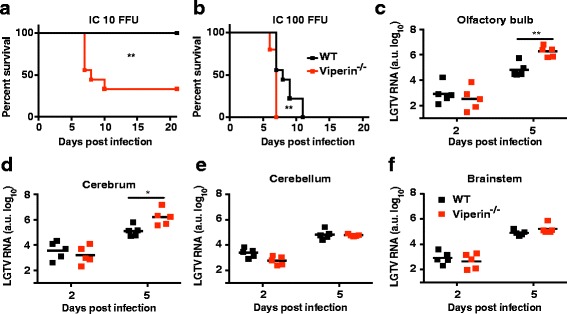
Viperin limits neurovirulence and control virus replication in the olfactory bulb and cerebrum. **a**–**b** Survival analysis of 6- to 8-week-old WT and viperin^−/−^ mice after intracranial inoculation of 10 (**a**) and 100 (**b**) FFU of LGTV (*n* = 10). Survival differences were tested for statistical significance by log-rank test. **c**–**f** Viral burdens in the brain regions (olfactory bulb, cerebrum, cerebellum, and brainstem) of WT or viperin^−/−^ mice infected intracranially with 100 FFU of LGTV (*n* = 5) were measured by qPCR at the indicated time points after infection. Bars indicated mean values. Asterisks indicate statistical significance calculated by the Mann-Whitney test (*P < 0.05; **P < 0.01)

To address the role of viperin in LGTV neurovirulence, mice were infected intracranially (ic) with a low (10 FFU) or a high (100 FFU) dose. At the low dose, all WT mice survived, whereas 60% of the viperin^−/−^ mice succumbed to infection (Fig. 3a). However, at the high dose, both WT and viperin^−/−^ mice succumbed to infection. Viperin^−/−^ mice died earlier, within 7 days compared to 11 days for WT mice (Fig. 3b). Thus, viperin provides a dose-dependent protection against LGTV neurovirulence. To correlate the enhanced susceptibility of the viperin^−/−^ mice to the regional differences in LGTV replication, virus replication was monitored in different brain regions after intracranial infection at the high dose. The olfactory bulb, cerebrum, cerebellum, and brain stem were harvested at 2 and 5 dpi, then analyzed for viral replication using qPCR. No difference could be detected in the viral burden between WT and viperin^−/−^ mice at 2 dpi. However, at 5 dpi substantially higher viral RNA levels were found in the olfactory bulb and cerebrum of viperin^−/−^ mice, while no differences were observed in the cerebellum and brain stem (Fig. 3c–f). These results suggest that viperin functions to suppress neurovirulence by restricting viral replication within the olfactory bulb and cerebrum.

### Robust induction of viperin after infection restricts viral growth in the cerebral neurons and astrocytes

**Fig. 4:**
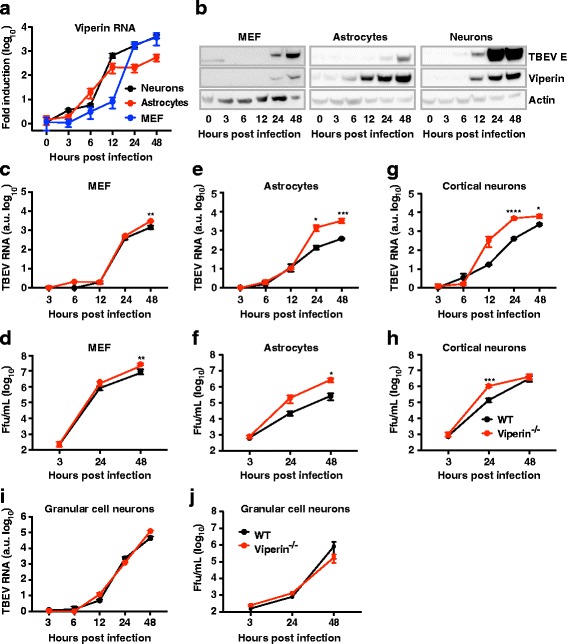
Viperin is induced in primary mouse embryonic fibroblasts, astrocytes, and cortical neurons after TBEV infection and inhibits viral replication. Primary MEFs, cortical neurons, and cortical astrocytes were isolated and differentiated from WT and viperin^−/−^ mice and infected with TBEV (MOI, 0.1) (**a**–**f**). **a** Viperin RNA upregulation in cells isolated from WT mice and infected by TBEV was measured by qPCR at the indicated time points. Viperin mRNA was normalized to the GAPDH expression, and to uninfected control. **b** Viperin protein expression from the same set up as in **a** was determined at the indicated time points by western blot. Viral replication kinetics in cells isolated from WT and viperin^−/−^ mice was determined at indicated time points by qPCR (**c**, **e**, **g**) and focus-forming assay (**d**, **f**, **h**). Primary granular cell neurons were isolated from the cerebellum of WT and viperin^−/−^ mice, differentiated, and infected with TBEV (MOI, 0.1). Viral replication was assayed by qPCR (**i**) and production of progeny virus by FFA (**j**). Viral RNA was normalized to the cellular expression of GAPDH, and the viral input 3 hpi was set to 1. Statistical significance was calculated using unpaired *t* test, significance is indicated by asterisks (*P < 0.05, ***P < 0.001, ****P < 0.0001). Data are cumulative from at least two independent experiments performed in triplicates

Since we found that viperin was able to restrict LGTV in vivo*,* we next wanted to determine the role of viperin in response to highly pathogenic TBEV infection (Biosafety level 3). To identify the brain cell types in which viperin functions to restrict the spread of the virus through the cerebrum, we used primary cultures of neurons and astrocytes. First, the expression of viperin was analyzed after TBEV and LGTV infection in astrocytes and cortical neurons isolated from the cerebrum of WT mice. As a control, primary mouse embryonic fibroblasts (MEFs) were used in which the type I IFN response has been well characterized (39–42). Cell cultures were infected with either LGTV or TBEV (MOI 0.1) and analyzed at different time points post-infection for viperin RNA, protein expression, as well as for viral replication. All cell types were able to upregulate viperin upon LGTV and TBEV infection (Fig. 4a and data not shown). The upregulation kinetics were similar for both neurons and astrocytes, while MEFs show a delayed kinetics of viperin RNA (Fig. 4a). However, uninfected astrocytes show 22-fold higher baseline RNA level of viperin compared to neurons (Additional file 2: Figure S2A). In line with this, low levels of viperin protein were detected before infection in astrocytes but not in neurons. Viperin protein levels increased robustly from 6 hpi in astrocytes following TBEV infection. In neurons, viperin reached the detection threshold at 12 hpi (Fig. 4b). A weak and delayed viperin protein expression was observed in MEFs.

To investigate the protective role of viperin in these cell types, replication kinetics were examined in WT and viperin-deficient cells, following quantification of viral RNA replication by qPCR (Fig. 4c, e, g) and viral infectious progeny (titers) using focus-forming assays (FFA) (Fig. 4d, f, h and Additional file 2: Figure S2). No differences in viral RNA replication were observed between WT and viperin^−/−^ MEFs. However, the viral titer was significantly higher in the absence of viperin at 48 hpi, which correlated well with the kinetics of viperin protein expression (Fig. 4b–d). A strong growth advantage of LGTV and TBEV was seen in the absences of viperin in both astrocytes and cortical neurons (Fig. 4e–h and Additional file 2: Figure S2B, C).

Since viperin was not necessary to restrict viral replication in the cerebellum in vivo, the granule cell neurons were isolated from the cerebellum of WT and viperin^−/−^ mice and infected with of TBEV, and the infection was monitored over time. No difference in growth was observed in the absence of viperin (Fig. 4i, j), indicating that neurons might play a role in the region-specific dependence of viperin in the brain. Overall, our results establish that viperin is induced in neurons and astrocytes after TBEV infection and serves as an important antiviral ISG in controlling TBEV infection.

### Viperin is needed for the IFN-mediated antiviral responses in the cortical neurons and astrocytes

**Fig. 5:**
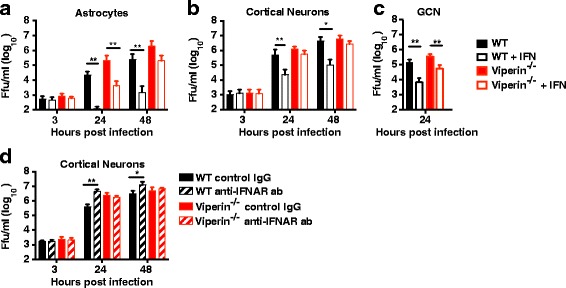
Type I IFN treatment can partly compensate for lack of viperin in astrocytes but not in neurons. Primary WT and viperin^−/−^ mice astrocytes (**a**) and cortical neurons (**b**) and granule cell neurons (GCN, **c**) were infected with TBEV (MOI, 0.1), and viral growth was determined at indicated time points. Sixteen hours before infection, astrocytes and cortical neurons were treated with 5000 U/ml IFNαB/D and virus titers were determined by focus-forming assay at the indicated time points. **d** Primary cortical neurons isolated from WT and viperin^−/−^ mice were treated with 10 μg/mL IFNAR-blocking antibodies or isotype control for 2 h, infected with TBEV (MOI, 0.1), and viral growth was determined by focus-forming assay at indicated time points. Statistical significance was calculated using unpaired *t* test, significance is indicated by asterisks (*P < 0.05, **P < 0.01). The results are cumulative of at least two independent experiments performed in triplicates

We have previously shown that the type I IFN response is important to protect against TBEV infection in mice and primary astrocytes [3, 4]. Since the IFN response involves hundreds of genes, we wanted to see if other ISGs upregulated after IFN treatment could compensate for the loss of viperin. Primary astrocytes, cortical neurons, and granular cell neurons from WT and viperin^−/−^ mice were pretreated with recombinant human IFNαB/D [37]. Viral growth kinetics was analyzed after TBEV infection (Fig. 5a–c). Pretreatment with IFN strongly reduced TBEV infection of WT astrocytes and both types of neurons indicating that they are very responsive to IFN. In contrast, IFN treatment of viperin^−/−^ astrocytes only partly reduced TBEV titers at 24 hpi, while no significant reduction was observed at 48 hpi. This indicates that viperin is a key factor in the antiviral IFN response in astrocytes. Although comparable levels of antiviral ISGs (Mx1, CXCL10, and Ifit1) were upregulated after IFN treatment of WT and viperin^−/−^ cortical neurons (Additional file 3: Figure S3), the IFN treatment of viperin^−/−^ cortical neurons failed to inhibit TBEV infection, independent on the time point analyzed, suggesting that viperin is the main antiviral ISG against TBEV in the cortical neurons. Interestingly, IFN treatment of the granule cell neurons isolated from viperin^−/−^ mice strongly reduced viral growth (Fig. 5c), indicating that the response upregulated after IFN differ between neuronal subtypes.

To test whether the robust upregulation of viperin after virus infection (Fig. 4a, b) in response to IFN in cortical neurons is functional, the IFNAR was blocked with monoclonal antibodies. WT and viperin^−/−^ neurons were treated with anti-IFNAR, or control antibody and infected with TBEV. In WT cortical neurons, blockage of IFNAR resulted in increased TBEV titers at 24 and 48 hpi (Fig. 4d). However, in viperin^−/−^ cortical neurons, IFNAR blockade had no impact on TBEV titer, further supporting a role for viperin as the main antiviral ISG active against TBEV in cortical neurons (Fig. 5d).

**Fig. 6:**
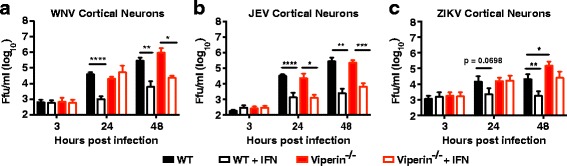
Viperin is an important ISG to control ZIKV infection in cortical neurons**.** Primary cortical neurons isolated from WT and viperin^−/−^ mice were infected with WNV (**a**), JEV (**b**), or ZIKV (**c**) (MOI, 0.1), and viral growth was determined by focus-forming assay at indicated time points. Subsets of neurons were treated with 5000 U/ml IFNαB/D for 16 h prior to infection. Statistical significance was calculated using unpaired *t* test, significance is indicated by asterisks (*P < 0.05, **P < 0.01). The results are cumulative of at least two independent experiments performed in triplicates

Numerous flaviviruses have been shown to be sensitive to the antiviral action of viperin [16, 18, 19, 21]. However, most studies have relied on overexpression of viperin in different cell culture systems, and not in primary cells that endogenously induce viperin. Therefore, the role of viperin as an ISG was tested for other neurotropic flaviviruses in the primary cortical neurons (Fig. 6a–c). Two viruses known to be sensitive to viperin, WNV and ZIKV [18, 21], and one known to be resistant to the antiviral activity, JEV [40], were chosen. Pretreatment of WT neurons with IFN strongly reduced the infection of all three viruses (Fig. 6a–c), showing that cortical neurons can respond to IFN treatment and mount an antiviral response against all tested viruses. No difference in growth pattern was detected between WT and viperin^−/−^ neurons independent on IFN treatment after JEV infection (Fig. 6b), supporting previously published data that JEV is resistant to the antiviral activity of viperin [40]. However, as seen for TBEV infection, no antiviral effect after IFN treatment of viperin^−/−^ cortical neurons was detected on both WNV and ZIKV infection 24 hpi (Fig. 6a, c). Interestingly, WNV infection was significantly reduced after 48 hpi in viperin^−/−^ cortical neurons treated with IFN, indicating that other ISGs also contribute to the IFN-mediated antiviral responses against WNV.

In summary, we have shown that viperin mediates region- and cell-type-specific inhibition of different neurotropic flaviviruses.

## Discussion

Viperin is a key player in the IFN-mediated antiviral response and inhibits a broad spectrum of viruses at various stages of their life cycles [41, 42]. Previously, we have shown that ectopic expression of viperin strongly inhibits TBEV and ZIKV replication by inhibiting viral genome synthesis by degrading viral NS3 [19, 28]. Here we used LGTV, as a surrogate model virus to determine the protective antiviral role of viperin in neurotropic flavivirus infection in vivo. We show that the antiviral effects of viperin are restricted to the specific brain regions, olfactory bulb and cerebrum, and this is probably mediated by their resident neurons. Finally, we also show that IFN-induced ISGs fail to compensate for the loss of viperin in the cortical neurons against various neurotropic flaviviruses.

Few studies have sought to define the function of viperin in vivo. For WNV, higher viral burdens were observed in the spleen, kidney, and brain in the absence of viperin, which was evident as viperin^−/−^ mice were more susceptible to infection [18]. Although both WT and viperin^−/−^ mice survived LGTV infection, we detected higher early viral burden in the spleen, before the olfactory bulb and then cerebrum in viperin^−/−^ mice. Thus, the spleen could serve as an organ for early viral amplification. Viral replication and subsequent spread within the brain is an important concern in neurotropic viral infection as it results in subsequent morbidity and mortality. Our results show that viperin reduces LGTV dissemination into the brain and limits the subsequent replication in the cerebrum. Previous studies using vesicular stomatitis virus (VSV) have shown that astrocytes within the olfactory bulb produce an IFN response, which restricts VSV infection. It induces long-distance IFN signaling within the brain, which activates antiviral ISG expression in distinct brain parts that prevents virus spread within the brain [43–45]. We found that although LGTV elicits an IFN response in the olfactory bulb of WT and viperin^−/−^ mice, LGTV disseminates into the cerebrum but not to the cerebellum in the absence of viperin. In addition, particularly cortical neurons rely on viperin to restrict viral growth in the presence of IFN, whereas granular cell neurons in the cerebellum can mediate an antiviral response in the absence of viperin. Our results indicate that the long-distance IFN signaling and antiviral response is blunted against LGTV in the absence of viperin.

There are conflicting studies regarding the role of viperin in interferon production. One study showed that viperin enhances TLR-7 and TLR-9 signaling and facilitates the nuclear translocation of IRF-7 to induce type I IFN response in plasmacytoid dendritic cells (pDCs) [39], while another study in macrophage cells showed that viperin acts as a negative regulator of an interferon response by interacting with interferon-beta promoter stimulator 1 (IPS-1) [46]. We found that viperin was involved in the systemic IFN response after LGTV infection; similar findings were observed in WNV infection [18]. In contrast, viperin was not involved in the IFN induction in the olfactory bulb. Most likely this reflects the cell-type-specific role of viperin in regulating the IFN response, since viperin is needed for the TLR-7/9 mediated induction in pDCs, and pDCs are known for their capacity to produce large amounts of type I IFN [47, 48], whereas IFN induction in the olfactory bulb is strongly dependent on IPS-1 [2].

Recent findings suggest differential immune responses within distinct regions of the brain that can modulate virus pathogenesis [2, 38, 49]. In our system, we observed a region-specific role of viperin following ic LGTV infection with higher viral replication in the olfactory bulb and cerebrum of viperin^−/−^ mice. This was consistent with the observations that viperin inhibited LGTV in the olfactory bulb and cerebrum after peripheral viral replication. In WNV and VSV infection, an antiviral effect of IFIT2 has been observed in the olfactory bulb, cortex, cerebellum, and brain stem [50, 51]. After WNV infection in Ifi27la^−/−^ mice, regional restriction was observed in the brain stem and cerebellum in the context of peripheral viral infection, while only in the brain stem post-ic infection [5], while viperin was required to control WNV replication in cortex and white matter [18], demonstrating distinct roles of ISGs, in different brain regions. This phenomenon might be due to the different subsets of neurons in different regions, as cortical neurons rely on viperin expression to limit TBEV replication while granule cell neurons isolated from cerebellum do not. Both epigenetic- as well as microRNA-mediated mechanisms might be involved in the regulation of ISGs in different subsets of neurons [38]. However, further studies are required to understand the complexity and heterogeneity of the ISG response within different brain regions. We speculate that cell-type- and tissue-specific differential expression of these ISGs within different brain regions and the availability of cofactors might influence their antiviral action.

Astrocytes are important producers of IFNs during neurotropic viral infections [43, 52]. We have previously shown that IFN signaling controls flavivirus infection and that viperin is highly upregulated in astrocytes [4]. Here we could demonstrate the importance of viperin in astrocytes, and that it acts in concert with other ISGs to restrict TBEV infection. One such candidate ISG is TRIM79α which is also highly upregulated in astrocytes [4], and antivirally active against TBEV [53]. Other ISGs like ISG15, Oas1b, Ifit2, PKR, and RNase L were also upregulated in astrocytes after TBEV infection and might also contribute to the antiviral effects against TBEV in the absence of viperin [4].

Neurons are the main target of TBEV infection in the CNS. We have previously shown that both WT and in the absence of IPS-1 or IFNAR signaling, neurons are the main target of LGTV infection [2, 3]. Cortical neurons induce type I IFN after infection, respond to IFN treatment, and upregulate viperin and other ISGs (Mx1 and Ifit1). However, here we show that they rely on viperin expression to reduce TBEV and ZIKV replication, indicating that neither Mx1 nor Ifit1 is targeting TBEV replication. We and others have found that endogenous viperin expression fails to control WNV in cortical neurons [18]. However, we now show that viperin is needed for the early IFN-mediated antiviral responses against WNV in these neurons. A possible explanation could be that WNV efficiently inhibits viperin induction in cortical neurons and thereby avoid its antiviral effect. WNV interferes with both upregulation of IFN through RIG-I-IPS-1 and cGAS-STING signaling pathways as well as inhibit IFNAR-mediated signal transduction (reviewed in [54]). The different impact of viperin on the different flaviviruses could also be explained by differences between the flaviviruses.

## Conclusions

In summary, we have found that viperin is important in specific regions of the brain to control virus replication. This dependency on viperin was further mapped to neuronal subtypes. We found that viperin was the most important ISG against TBEV and ZIKV in the cortical neurons, as IFN treatment could not compensate for the loss of viperin. Similar but not as dramatic effect was also found for WNV. These findings suggest that a single ISG can shape the susceptibility and immune response within different regions of the brain.

## Additional files

Additional file 1: **Figure S1.** Intraperitoneal LGTV infection does not alter the body weight of WT or viperin^−/−^ mice. 6- to 8-week-old WT and viperin^−/−^ mice were inoculated with 10^4^ FFU of LGTV by intraperitoneal route (*n* = 10). Body weight was measured daily for 21 days. (PDF 503 kb)

Additional file 2: **Figure S2.** Viperin is highly upregulated in astrocytes after TBEV infection, and it inhibits LGTV infection in cortical astrocytes and neurons. Primary cortical neurons and cortical astrocytes were isolated and differentiated from WT mice and infected with TBEV (MOI, 0.1). (A) Viperin expression was measured by qPCR at the indicated time points. Viperin mRNA was normalized to the GAPDH expression and represented as dCt. Primary WT and viperin^−/−^ astrocytes (B) and cortical neurons (C) were infected with LGTV (MOI, 0.1), and viral growth was determined at indicated time points by focus-forming assay. Statistical significance was calculated using unpaired *t* test, significance is indicated by asterisks (**P < 0.01, ***P < 0.001). Data are cumulative from at least two independent experiments performed in triplicates. (PDF 541 kb)

Additional file 3: **Figure S3.** Viperin is not needed for the induction of ISGs in cortical neurons and astrocytes. Primary WT and viperin^−/−^ mouse cortical neurons and astrocytes were treated with 5000 U/mL IFNαB/D for 16 h or left untreated. mRNA expression of Mx1, IFIT1, and CXCL10 was determined by qPCR. Expression levels were normalized to the endogenous GAPDH expression and depicted as mRNA arbitrary units (AU). (PDF 531 kb)

## Abbreviations

CNS: Central nervous system
FFU: Focus forming unit
hpi: Hours post-infection
IFN: Interferon
IFNAR: IFN-α/β receptor
IPS-1: Interferon-beta promoter stimulator 1
IRF: Interferon regulatory factor
ISG: Interferon-stimulated gene
JAK: Janus kinase
JEV: Japanese encephalitis virus
LGTV: Langat virus
MEF: Mouse embryonic fibroblast
qPCR: Quantitative reverse transcription PCR
STAT: Signal transducer and activator of transcription
TBEV: Tick-borne encephalitis virus
VSV: Vesicular stomatitis virus
WNV: West Nile virus
WT: Wild-type
ZIKV: Zika virus
