# Supplementary figures and images for: Cell-type- and region-specific restriction of neurotropic flavivirus infection by viperin
Source: J Neuroinflammation. 2018 Mar 15;15:80. doi: 10.1186/s12974-018-1119-3 (PMC5856362; doi:10.1186/s12974-018-1119-3)

Figure S1

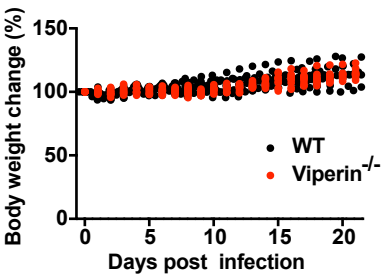

Supplement: Supplementary file 1 — Figure S1. Intraperitoneal LGTV infection does not alter the body weight of WT or viperin−/− mice. 6- to 8-week-old WT and viperin−/− mice were inoculated with 104 FFU of LGTV by intraperitoneal route (n = 10). Body weight was measured daily for 21 days. (PDF 503 kb) [file 12974_2018_1119_MOESM1_ESM.pdf]

Figure S2

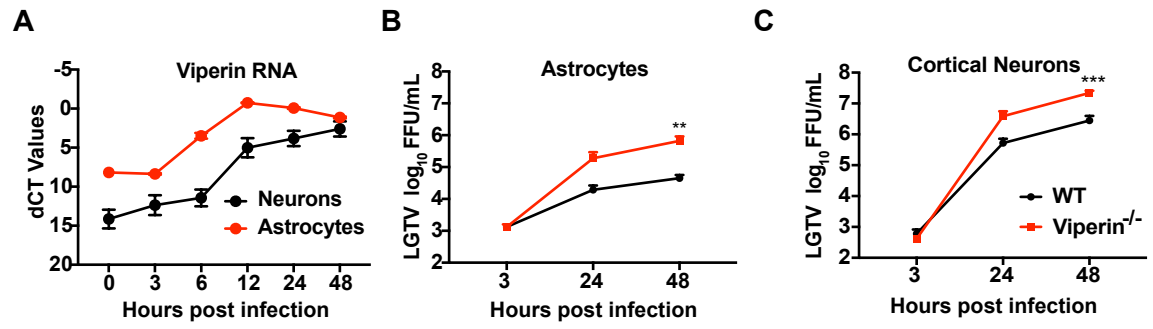

Supplement: Supplementary file 2 — Figure S2. Viperin is highly upregulated in astrocytes after TBEV infection, and it inhibits LGTV infection in cortical astrocytes and neurons. Primary cortical neurons and cortical astrocytes were isolated and differentiated from WT mice and infected with TBEV (MOI, 0.1). (A) Viperin expression was measured by qPCR at the indicated time points. Viperin mRNA was normalized to the GAPDH expression and represented as dCt. Primary WT and viperin−/− astrocytes (B) and cortical neurons (C) were infected with LGTV (MOI, 0.1), and viral growth was determined at indicated time points by focus-forming assay. Statistical significance was calculated using unpaired t test, significance is indicated by asterisks (**P < 0.01, ***P < 0.001). Data are cumulative from at least two independent experiments performed in triplicates. (PDF 541 kb) [file 12974_2018_1119_MOESM2_ESM.pdf]

Figure S3

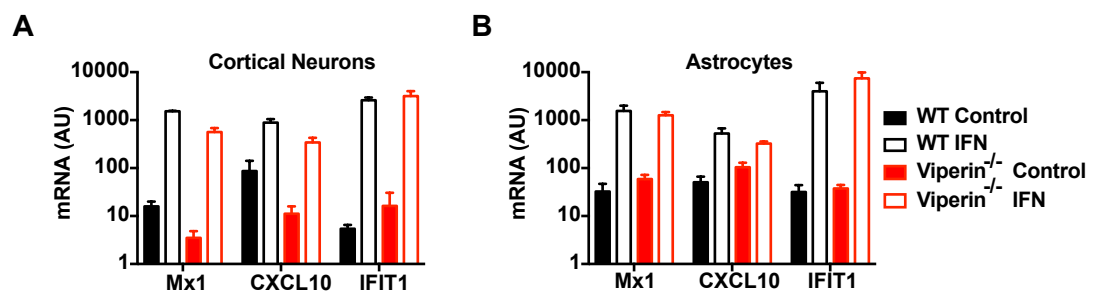

Supplement: Supplementary file 3 — Figure S3. Viperin is not needed for the induction of ISGs in cortical neurons and astrocytes. Primary WT and viperin−/− mouse cortical neurons and astrocytes were treated with 5000 U/mL IFNαB/D for 16 h or left untreated. mRNA expression of Mx1, IFIT1, and CXCL10 was determined by qPCR. Expression levels were normalized to the endogenous GAPDH expression and depicted as mRNA arbitrary units (AU). (PDF 531 kb) [file 12974_2018_1119_MOESM3_ESM.pdf]
